# Strangers in a Strange Land: Relations Between Perceptions of Others' Values and Both Civic Engagement and Cultural Estrangement

**DOI:** 10.3389/fpsyg.2019.00559

**Published:** 2019-03-22

**Authors:** Rebecca Sanderson, Mike Prentice, Lukas Wolf, Netta Weinstein, Tim Kasser, Tom Crompton

**Affiliations:** ^1^Common Cause Foundation, Powys, United Kingdom; ^2^Department of Psychology, Wake Forest University, Winston Salem, NC, United States; ^3^School of Psychology, Cardiff University, Cardiff, United Kingdom; ^4^Department of Psychology, University of Bath, Bath, United Kingdom; ^5^Department of Psychology, Knox College, Galesburg, IL, United States

**Keywords:** values, cultural estrangement, civic engagement, voting, institutions

## Abstract

Healthy democracies require civic engagement (e.g., voting) from their citizens. Past research has suggested that civic engagement is positively associated with self-transcendence values of care and concern for the welfare of others, and negatively associated with self-enhancement values of self-interest, dominance, and personal success. However, research has yet to address whether people's *perceptions of others' values* are related to civic engagement. Across three studies with nationally representative samples in the UK and US (*N*s ≥ 1,000), we explored how civic engagement relates to (a) perceptions of national values, (b) perceptions of the values of one's typical compatriot, and (c) perceptions of the values encouraged by social and cultural institutions. Study 1 showed that the tendency for British citizens to perceive British culture as valuing self-transcendence was associated with an increased likelihood of voting in the 2015 general election. These findings were replicated for “a typical British person” (Study 2) and “a typical American person” (Study 3); Studies 2 and 3 also found that perceived self-enhancement values of typical compatriots were negatively correlated with reported voting. We also examined how perceptions of others' values relate to cultural estrangement—the feeling of not fitting in one's culture or of being atypical. Like civic engagement, those who perceived less self-transcendence and more self-enhancement in their culture felt more culturally estranged. Mediation analyses in Studies 2 and 3 revealed that estrangement helped to explain the relationship between perceptions of others' values and voting. In sum, the extent to which Brits and Americans perceive that self-transcendence values are strongly held by other citizens is associated with feeling less estranged and with reports of being more civically engaged. In contrast, the perception that these targets hold or promote self-enhancement values is positively associated with feelings of estrangement, to the detriment of civic engagement. Implications for future research and democratic processes are discussed.

## Introduction

“*Americans today continue to have a lot in common and share many values, beliefs, and attitudes. Yet most Americans do not feel this way; most perceive a crisis of values.”* Baker (2005, p. 110)

“*Here I am the barbarian, because I am not understood by anyone.”* Rousseau (1751, p. 1)

The experience of not belonging, of looking in disbelief at one's compatriots and wondering whether there can be common ground, may be familiar to UK and US citizens in the contentious run-ups to and aftermaths of the 2016 EU referendum and the 2016 Trump-Clinton presidential race. During such significant votes, public discourse often turns to the topics of values and national identity. Questions like “Why are other people voting for this?” and “What does our nation really care about right now?” are frequently asked. As Baker's and Rousseau's quotations above suggest, people tend to have ideas about what their compatriots value. The purpose of the present research is to examine whether these perceptions of others' values bear any relations to whether people feel they belong in their culture and whether they turn up at the polls to vote or engage in other forms of civic participation.

In so doing, this research provides a novel glimpse into how perceptions of others' values relate to civic engagement. Much has been written on how civic engagement relates to personal values (Schwartz, 2010; Pacheco and Owen, 2015; Vecchione et al., 2015), but not, to our knowledge, on how it connects to perceptions of others' values. In addition to exploring the relationship between perceptions of others' values and civic engagement, we also explore the possibility that this relationship is mediated by cultural estrangement, a variable that past research has related to discrepancies between personal and societal values (Bernard et al., 2006) and to civic engagement (Hackett and Omoto, 2009).

We draw on the well-known theory of values developed by Schwartz (1992, 1994), which proposes a set of values that are likely to be universal because they derive from three broad requirements of human existence: People's needs to function as biological organisms, to take part in coordinated social interaction, and to look after the survival and welfare of their groups. Schwartz's values theory yields a set of higher-order values, two of which, self-transcendence and self-enhancement, have been shown in previous research to have particularly clear and consistent relationships with a wide range of social and environmental outcomes (Strauss et al., 2008; Crompton, 2010; Sagiv et al., 2011).

The self-transcendence/self-enhancement axis captures a conflict between opposing motivations: The self-transcendence values of care and concern for the welfare and interests of one's community and other people more generally, vs. the self-enhancement values of self-interest, personal success and dominance over others (Schwartz, 2012, p. 8). The theory holds that when self-transcendence values are held strongly by an individual at a particular moment, then it is likely, due to their motivational opposition, that self-enhancement values (and value-congruent behaviors) are temporarily suppressed (Maio et al., 2009). Such a “see-saw” action works in the opposite direction as well.

While all people are likely to care about both self-transcendence and self-enhancement values to some degree (Bardi and Schwartz, 2003), the relative priority people place on them is an important correlate of civic outcomes. Compared to focusing on self-enhancement values, valuing self-transcendence at high levels (and self-enhancement at low levels) is associated with relatively less prejudice, more acceptance of diversity (Strauss et al., 2008), more co-operation rather than competition (Sagiv et al., 2011), and more adoption of environmentally sustainable behaviors (Karp, 1996).

Civic engagement behaviors may be understood as “individual and collective actions designed to identify and address issues of public concern” (APA, 2016).^1^ Although the relationship between personal values and particular civic attitudes and behaviors will depend to some extent on political ideology (Boer and Fischer, 2013)–participating in a far right rally will be driven by different values than volunteering at a refugee camp, for instance, just as voting for a conservative candidate will be driven by different values than voting for a liberal candidate—there is also evidence that personally-held self-transcendence and self-enhancement values are relevant to broad classes of civic engagement activities. Several studies have found that, *regardless of political identification*, self-transcendence correlates positively, while self-enhancement correlates negatively, with activities such as participating in political demonstrations, contacting politicians, and signing petitions (Schwartz, 2010; Vecchione et al., 2015); participating in organizations to promote the welfare of people, animals, and the environment (Schwartz, 2010); helping others (Daniel et al., 2015); and volunteering locally (Sanderson and McQuilkin, 2017).

What about people's decision to not engage in civic matters, to not even use their right to cast a vote?

Over the last few decades, governments in both the UK and US have struggled with historically low voter turnout. For instance, Fink (2012) found that political participation fell further in the UK over the period 2002–2010 than in most other EU countries. Although there is scant research on the link between voter abstention and personal values, one study has noted that non-voters tended to be especially self-enhancement focused in their values (Caprara et al., 2012). In contrast, voters give comparatively high priority to values in the self-transcendence domain.

Given the established links between personal values and levels of civic engagement, it seems sensible to ask how people's beliefs about *others'* values relate to their civic engagement. When people decide to vote, campaign, or attend a public meeting, they are engaging with other citizens to influence civic outcomes as expressions of group values. The values perceived to be held by other people, therefore, are likely to bear some relation to an individual's decision to take part in civic life.

For example, if people believe that their neighbors generally prioritize self-transcendence values, then perhaps that belief acts in a number of ways as an incentive to show up to the polls. It could give people confidence that it is good to act in service of the group and to be able to report to others that one did the right thing; it might also give rise to thoughts like “If others care, then so should I.” If, on the other hand, people think their neighbors are motivated by self-enhancement, then perhaps this suppresses the desire to participate in matters of group concern. It could give people confidence that acting for group concern is a sucker's proposition, or, perhaps, give rise to thoughts like “Well everyone else is just out for themselves, so why should I bother?”

If, as Miller (1999) has argued, people in Western societies hold a norm of self-interest, then individuals might both assume that others are motivated to pursue their material self-interest and also acquiesce to that perceived, prevailing norm, i.e., adopt attitudes and enact behaviors consistent with the norm of self-interest. A perceived norm of self-interest might suppress civic engagement to the extent that such engagement is seen as primarily a group- (rather than self-) interested activity. A perceived norm of group-interest, on the other hand, might seem more congruent with civic activities, thereby encouraging individuals to acquiesce to perceived norms of engagement and become more involved. The influences of norms on behavior have been widely demonstrated (Cialdini et al., 1991); for example, interventions that involve giving information about the norms of a peer group can change drinking habits and environmentally-friendly behaviors (see Miller and Prentice, 2016).

The few studies that have examined perceptions of others' values have tended to focus on value stereotypes and the extent to which caricatures of a group's values are accurate or not. Dobewall and Strack (2011), for instance, found that various ethnic groups in Estonian society perceived all groups, including their own, as prioritizing self-enhancement values to a much greater degree than was actually reflected by European Social Survey data. Similarly, Brits assumed their compatriots were relatively lower on self-transcendence and higher on self-enhancement than was actually the case (Bernard et al., 2006), and Russian participants reported believing “typical Russians” to be more self-enhancement oriented than they were themselves (Lönnqvist et al., 2012). A common conclusion emerges from this small literature: People often perceive value differences between themselves and their compatriots, typically such that their compatriots are believed to be more self-enhancement oriented than they are themselves.

Having observed these (mis)perceptions in several cultures, we wondered whether they have any systematic relations to civic engagement behaviors. Some evidence supports the idea that people's behavior may be affected by the assumptions they have about other people's values.

For instance, one study found that perceptions of the values of out-group members relate to out-group antagonism or altruism: In a resource allocation task, both German and Israeli participants allocated more resources to their “out-group” (Israelis and Germans, respectively), the more they perceived them to value self-transcendence (Schwartz et al., 1990). In another study, participants were presented with the Prisoner's Dilemma Game, but it was named either the “Wall Street Game” or the “Community Game” (Liberman et al., 2004). Wall Street Game players were consistently more likely to betray the other players and attempt to win the highest reward compared to those who played the Community Game, who were more likely to cooperate. The authors suggested that the name of the game affected people's expectations regarding how others would play, which then changed how they themselves played. If these processes also occur at the civic level, then citizens living in what they believe to be a caring and co-operative environment (i.e., where others prioritize self-transcendence values) could be more likely to participate in a caring and cooperative way than those who believe they exist in a self-interested environment (i.e., where others prioritize self-enhancement values).

These reflections led us to formulate our first two hypotheses.

**H1a & H1b:** Civic engagement will be (a) positively correlated with perceptions that other people value self-transcendence and (b) negatively correlated with perceptions that other people value self-enhancement.

Following the results of studies on perceived value discrepancy, we also expect that perceptions of values may be associated with cultural estrangement. The word estrangement comes from the Latin verb *extraneare*, to treat as a stranger. Thus, people who experience estrangement feel like a stranger to the people or society around them. Estrangement is typically understood specifically as the rejection of, or removal from, the dominant values in society (Cozzarelli and Karafa, 1998). It has been situated as a vital component within alienation (Bernard et al., 2006, p. 78), a broader construct studied deeply in sociology and philosophy that concerns a more multi-faceted dissociation than merely from society's values (e.g., from the production of goods).

Bernard et al. (2006) showed that people felt more estranged the more of a discrepancy there was between their personal values and the ratings of those same values in British society. Estrangement was particularly high amongst people who felt like society cared less about the welfare of others than they did personally.

Such results lead us to the reasoning that cultural estrangement may be driven by beliefs about others' values *per se*. Self-enhancement values are by definition self-oriented rather than group-oriented, so it is plausible that believing that other people prioritize selfish goals generates feelings of not fitting in and not being included in other people's concerns, whatever one's own value priorities. We reasoned that, in contrast, believing one's welfare is included in others' spheres of concern (even via mere citizenship) would be negatively associated with the subjective experience of being estranged. We therefore formulated these next two hypotheses.

**H2a and H2b:** Cultural estrangement will be (a) negatively correlated with perceptions that others value self-transcendence and (b) positively correlated with perceptions that others value self-enhancement.

While there is very little research on the relationship between cultural estrangement and political engagement, some evidence suggests that it is negative. Hackett and Omoto (2009) found that estrangement after an election was significantly higher in non-voters than in voters; it was also associated with low political efficacy (i.e., the belief that one has the competence and skills to engage with politics) and low intention to become politically engaged in future (e.g., to vote, to protest, to work in the community). This finding corresponds with literature that describes alienation as deriving, in part, from feelings of isolation and being ineffective in the world (Stokols, 1975). We therefore formed hypothesis H2c.

**H2c**: Cultural estrangement will be negatively correlated with civic engagement.

We reasoned, given these hypotheses, that estrangement might explain the association between perceptions of others' values and civic engagement. If people believe their compatriots to be selfish, for instance, it could be the corresponding feeling of disconnection from dominant values that explains why they choose not to get civically involved. Put in mediation analytical terms, we hypothesize that perceptions of others' values will be related to estrangement (the *a* path in Baron and Kenny, 1986) and civic engagement (the *c* path), that estrangement will be related to civic engagement (the *b* path), and that estrangement will explain (as the *ab* product) the relations between perception of others' values and civic engagement [the [reduced] *c'* path].

**H2d:** Cultural estrangement will mediate the relationship between perception of others' values and civic engagement.

In addition to testing the hypotheses above, we sought to explore how cultural estrangement and civic engagement might relate to people's perceptions of the values promoted by cultural institutions. It is partly through having contact with the rules, practices, and norms of institutions that people obtain a sense of the values that are held to be important in their culture, as well as the values that are encouraged in them as citizens (Schwartz and Sagie, 2000).

Although the values of both institutions and other people can be considered indicators of what society cares about, we reasoned that institutions tend to be more reflective of the values of prevailing ideologies, such as neoliberalism or capitalism, that may go beyond more day-to-day concerns of oneself and compatriots. The value priorities of citizens have been shown to relate to the extent to which their country's economy is actually organized according to de-regulated, free-market capitalist principles (Kasser et al., 2007; Kasser and Linn, 2016). But do the values people *think* are enshrined in and promoted by their institutions relate to civic (dis)engagement? To our knowledge, such questions have not been studied.

Perhaps the most relevant literature to this question is that concerning values in the workplace which has shown that people who believe that their workplace values altruism and relationships (similar to self-transcendence values: Ros et al., 1999; Cable and Edwards, 2004) are more satisfied with their job and have greater intention to stay than do those who do not see such values as prioritized in the workplace (Edwards and Cable, 2009).

Admittedly, it is a leap to infer that workplace dynamics parallel those of large national institutions, but insofar as people can attribute values to abstract entities (like companies) and engage differently depending on these attributions, a similar pattern might hold in the civic sphere. If that is so, we expect that the pattern of relations between perceptions of institutional values and civic engagement will be similar to the pattern of relations between perceptions of other people's values and civic engagement specified above, as institutions also play a role in signaling what values are important in society and how individuals should therefore behave.

**H3a and H3b:** Civic engagement will be (a) positively associated with how much people perceive institutions to encourage self-transcendence and (b) negatively associated with how much people perceive institutions to encourage self-enhancement.

Finally, we explored whether perceptions of institutional values would relate to cultural estrangement. If cultural estrangement entails the experience of feeling separate from culture, and people look to institutions to get a sense of cultural values, then we imagine, for the reasons stated above (H2a), that the perceived encouragement of self-transcendence might correlate with lower estrangement, while self-enhancement might correlate with higher estrangement.

## The Present Research

We conducted three studies that examine the relations among perceived values for self-transcendence and self-enhancement and how much people are civically engaged, have positive attitudes about civic engagement, and report feeling estranged from their cultures. Study 1 asked British residents to report on the values essential to being British (i.e., “what it means to be British”) and whether they voted in recent elections. Next, representative samples of Brits (Study 2) and Americans (Study 3) reported on the values of a “typical Brit/American” and whether they (a) voted in local and national elections, (b) participated in other (non-voting) forms of civic engagement (like signing petitions), (c) held positive attitudes about civic engagement, and d) felt culturally estranged. In addition, participants in Studies 2 and 3 indicated the extent to which they believed various cultural institutions, like the government and the media, promoted self-transcendence and self-enhancement.

Readers might expect that we tested our main hypotheses after controlling for ratings of personally- held self-enhancement and self-transcendence values. We did not do so, however. Please refer to Appendix for our reasons.

## Study 1

### Material and Methods

#### Participants

One thousand seven hundred and sixty-two participants (51.1% women, 48.9% men; 18–91 years of age, *M*_age_ = 48.42, *SD* = 16.56; 88.1% White British; 80.3% born in the UK) took part in an online survey administered by market research organization YouGov in Spring of 2015. Participants took ~12 min on average to complete the survey and received credits from YouGov that they could exchange for shopping vouchers or money. Participants who did not complete all measures were excluded from relevant analyses, leaving a maximum *N* = 1,045.

#### Procedure

After giving their informed consent to take part in the study, participants were asked to indicate their age, gender, ethnicity, gross household income, country of birth, and which political party they identified with. Next, participants answered the question “To what extent do you consider yourself as British?” on a scale from 1 (*not at all*) to 5 (*completely*), with additional options to answer ‘*don’t know'* or to skip the question. To measure participants' perception of which values are important in British culture, they were asked “Now we'd like you to think about British culture and identity. To what extent do you agree or disagree that the following statements summarize an important part of what it means to be British: Pursuing authority, wealth, ambition, and influence” (an indicator of self-enhancement); a similarly worded question replaced these value elements with “Valuing equality and peace, being honest and friendly” (an indicator of self-transcendence). Participants were also presented with three statements about their feelings about being British, as these were all candidate correlates of voting behavior: “I feel good about being British,” “I feel alienated from British culture,” and “I care about the welfare of Britain.” In response to all five statements, participants indicated how strongly they agree from 1 (*strongly agree*) to 5 (*strongly disagree*), with the options to answer ‘*don’t know'* or to skip the question. Responses to these five questions were re-coded prior to analysis so that higher scores indicated feeling better about being British, believing Brits valued pursuing authority, wealth, ambition, and influence, etc.

Approximately 3 weeks later, within 3 days of the General Election, participants were asked “Talking to people about the General Election on May 7th, we have found that a lot of people didn't manage to vote. How about you? Did you manage to vote in the General Election?”^2^ Participants answered with ‘*yes*’ (59.9%), ‘*no*’ (5.6%), or ‘*don't know*' (0.3%), or did not respond at follow up (34.2%). Responses of ‘*don’t know'* were set to missing along with non-responses. Attrition analysis revealed, importantly, that perceived values did not predict responding vs. not responding (*p*s > 0.05). Demographics were explored as predictors of attrition, revealing negligible effects of age and household income, *b*s < 0.04, *p*s < 0.05. If participants answered yes to voting, they indicated which political party they had voted for, with the largest proportions going to Conservative (19.8%) and Labor (16.6%).

### Results

Our first hypothesis was that voting (i.e., our measure of civic engagement) would be positively related to believing that being British meant valuing self-transcendence and would be negatively related to believing that being British meant valuing self-enhancement. Analyses revealed that perceived self-transcendence values in British culture were positively related to voting, *r*_ST_ = 0.095, *p* = 0.002, but perceived self-enhancement values in British culture were not, *r*_SE_ = −0.003, *p* = 0.930. As shown in Table 1, all of the “British feelings” were significantly related to voting in the previous election. Further, all of the feelings were related to perceived self-transcendence in the predicted manner, but only feeling good about being British was related to self-enhancement (positively).

**Table 1:** Correlations and descriptive statistics for Study 1 variables.

	**Mean**	**SD**	**Voted**	**Feel Good**	**Alienated**	**Welfare**	**ST-Brit**
Voted	0.92	0.26					
Feel good	2.13	1.01	0.11^***^				
Alienated	3.64	1.12	−0.11^***^	−0.42^***^			
Welfare	1.63	0.70	0.13^***^	0.34^***^	−0.22^***^		
ST-Brit	2.87	1.07	0.16^***^	0.36^***^	−0.25^***^	0.28^***^	
SE-Brit	2.08	0.97	0.00	0.19^***^	0.02	0.04	0.06^*^

*** *p < 0.001*,

* p < 0.05. ST-Brit, Perceived importance of self-transcendence values for the “typical Brit.” SE-Brit, Perceived importance of self-enhancement values for the “typical Brit.”

Together, the correlational patterns suggested that all of the British feelings are candidate mediators that could help to explain the observed link between perceived self-transcendence values of others and voting. To examine this possibility, we conducted mediation analyses using Hayes' Process procedure (model 4, 5,000 bootstraps; Hayes, 2013). All models were logistic to appropriately model the yes/no voting variable. We conducted a series of analyses with one of the feelings entered at a time. The analysis with feeling good about being British revealed that feeling good was no longer a significant predictor of voting once perception of others' self-transcendence values was added to the model (*b* = 0.136, *SE* = 0.121, *z*_(1044)_ = 1.12, *p* = 0.261), and thus feeling good about being British was not a mediator of the relationship. In contrast, caring about Britain retained a significant effect on voting, *b* = 0.360, *SE* = 0.147, *z*_(1044)_ = 2.45, *p* = 0.014, and the CI for the indirect effect did not contain zero, *b* = 0.075, *SE* = 0.031, [0.015, 0.139], indicating mediation. Similarly, feeling alienated maintained a marginally significant prediction of voting in the presence of self-transcendence, *b* = −0.206, *SE* = 0.108, *z*_(1044)_ = −1.917, *p* = 0.055, and indirect effect analysis indicated that alienation was a mediator, *b* = 0.064, *SE* = 0.034, [0.002, 0.134]. Across all models, the perceived values maintained significant direct effects on voting despite the presence of the mediators. Mediation tests were not pursued for self-enhancement because it was not related to voting.

Overall, these analyses indicate that the relationship between participants' voting behavior and their perceptions of self-transcendence values in British culture is mediated by how much participants reported caring about the welfare of Britain and how little they feel alienated from British culture, but not by their satisfaction with being British.

### Brief Discussion

Study 1 provided initial support for the hypothesis that perceptions of others' self-transcendence values (in British culture) are positively tied to civic engagement (voting in the 2015 election). Further, this demonstration was prospective in that perceived values predicted subsequent voting behavior. Past studies (Schwartz, 2010; Vecchione et al., 2015) have shown that civic engagement is associated with one's own values, but, to our knowledge, Study 1's results are the first indication that perceptions of the content of others' values may be another important correlate of civic engagement. The current study also provides support for hypotheses 2a-c: The more that people perceived self-transcendence values to be prevalent among other Brits, the less they felt alienated, and, in turn, the more they reported voting. We did not find support, however, for the hypothesis that perceived self-enhancement values of other Brits would be negatively related to civic engagement, and thus mediational hypothesis testing was not conducted for this variable.

These results are of course preliminary and have (at least) one strong methodological limitation: Each construct was measured by a single item. It remains to be seen whether the present results might replicate with more thorough construct assessment and other measures of civic engagement.

Study 2 addresses each of these issues by employing expanded measures of perceived values, cultural estrangement, and civic engagement.

## Study 2

### Material and Methods

#### Participants

The sample was obtained in April 2015 by market research organization Ipsos MORI, which collected a representative sample of the British population in terms of age, gender, region of the UK, level of education, and income. The sample obtained had *N* = 1,000, 50% female. Age was binned by decade after a grouping that included 18–24 year olds, and every decade comprised at least 15% of the sample until 65–74 years and 75+ years. Participants received points for the Ipsos benefit system in exchange for completing the survey.

#### Overview of Procedure

Participants first completed informed consent and were assured of the confidentiality of their responses. Next, they completed demographic questions and a measure of socially desirable responding.^3^ Participants then completed a measure of values three times, once concerning themselves, once concerning the typical British citizen, and once concerning the values that they believe social/cultural institutions encourage them to hold. The order of the target rating (self, compatriot, and institution) was randomized across participants. Further, participants were randomly assigned to rate the values encouraged by one of five randomly assigned institutional targets: the government, the media, the education system, the business sector, and arts and culture. Next, participants completed a measure of civic engagement and then a measure of cultural estrangement. Finally, they were debriefed.

#### Measures

##### Self-enhancement and self-transcendence

The Personal Values Questionnaire-21 (PVQ-21; Schwartz, 2003) is a version of the PVQ-40 (Schwartz et al., 2001) shortened for the European Social Survey.^4^ It presents participants with 21 brief descriptions of people who give importance to one of the ten values in Schwartz's (1992) model. For instance, the description “It is important to him to be rich. He wants to have a lot of money and expensive things” reflects the value of power. Participants completed three versions of the PVQ, one for self, one for compatriot-as-target, and one for institution-as-target.

The prompt for the *self* was:

*Here we briefly describe some people. Please read each description and think about how much each person is or is not like YOU. Select the box underneath that shows how much the person in the description is or is not like YOU*.

Participants indicated to what extent each person was like them on a scale from 1 (*not like me at all*) to 6 (*very much like me*).

For the compatriot-as-target PVQ, the prompt replaced “YOU” with “A TYPICAL BRITISH CITIZEN.” The scale was anchored at 1 (*not at all like a typical British citizen*) and 6 (*very much like a typical British citizen*).

The prompt for *institutions* varied by the institution cited. For example, the prompt for the government institution condition read:

*We'd now like you to think about the GOVERNMENT in your country. Please read each description and think whether the GOVERNMENT in your country encourages you to be this kind of person. By the GOVERNMENT we mean the local authorities and the national and UK governments. Select the box underneath that shows how much you think the GOVERNMENT encourages you to be this kind of person*.

The scale for institution-as-target was anchored at 1 (*doesn't encourage me to be this kind of person at all*) and 6 (*very much encourages me to be this kind of person*).

The 10 value domain scores were first calculated as the mean of their two constitutive items (or of their three items, in the case of universalism). To investigate the relative importance of the values and control for some response biases, we subtracted the mean of all 21 PVQ items from the 10 value domain scores (i.e., ipsatized the scores; cf. Schwartz, 2003). These ipsatized domain scores were used in the computation of the higher-order scores and all subsequent analyses. For example, self-transcendence was the mean of the ipsative scores for benevolence and universalism.

Multidimensional scaling visualizations revealed that the compatriot and institution versions of the PVQ had a good fit to Schwartz's (1992) circumplex model of values for self-reports. That is, items that belonged to the higher order domains of self-transcendence and self-enhancement clustered together for the compatriot and institution versions in ways that would be expected from past analyses of the model that are based on self-reports of one's own values. Such results are consistent with other research regarding informant reports on the PVQ (e.g., in Polish samples, Skimina and Cieciuch, 2017). Thus, we proceeded to create the self-transcendence and self-enhancement scores for the compatriot and institution formats with the averaging and ipsatizing procedures described above.

Confirmatory factor analysis indicated that self and other values should be treated as separate constructs rather than attributed to the same latent construct. For example, for self-transcendence values in the UK sample, a model with two separate but covaried latent variables for the self and other items fit better [RMSEA = 0.051 [0.043, 0.059], SRMR = 0.033, CFI = 0.973, TLI = 0.965], than a single latent variable model for both self and other self-transcendence items [RMSEA = 0.196 [0.187, 0.205], SRMR = 0.133, CFI = 0.662, TLI = 0.565]. The superiority of separate construct models held across samples, value constructs, and combinations of self-, other-, and institution-targeted items.

##### Civic engagement—attitudes

The Civic Attitudes subscale of the Civic Engagement Scale (Doolittle and Faul, 2013) measures the feelings and beliefs people have about taking responsibility for and trying to make a difference in their community. Participants indicated their agreement with six statements, including “I feel responsible for my community” and “I believe that it is important to volunteer” on a scale from 1 (*strongly disagree*) to 7 (*strongly agree*). Two of the eight original items were dropped from the instrument prior to data collection because members of the research team suspected that they would not perform well in a British sample.^5^ The six remaining items had good internal reliability (α = 0.85).

##### Civic engagement—behaviors

To measure civic engagement behaviors, we asked whether participants had recently participated in 13 behaviors ranging from voting in local and national elections to getting in touch with government officials. These items were submitted to exploratory factor analysis using MLE and promax rotation. After dropping one poorly- and cross-loading item (about “clicktivism”), we noted a clear elbow in the scree plot after the second factor. The factors were interpretable in that one was clearly represented by *voting behavior* (the two items for local and national, mean standardized loading = 0.83) and the other captured *all the other forms of civic engagement behavior* (mean standardized loading = 0.51). We thus created two summary variables, one for voting and one for other civic engagement behaviors. Some civic engagement behaviors that constituted the *other forms* measure were signing petitions, volunteering for charities or campaigns, and donating money to organizations.

##### Cultural estrangement

The Cultural Estrangement Inventory (CEI, Cozzarelli and Karafa, 1998) is a ten-item questionnaire with two subscales. The “atypical” subscale measures the extent to which people think their values, beliefs and ideas differ from those of their compatriots (e.g., “I strongly identify with British values” reverse scored), and the “misfit” subscale measures the extent to which people feel like they do not fit in with the cultural mainstream (e.g., “People, on occasion, tell me that I am different.”). The CEI was developed for Americans, but it has previously been adapted for British participants (e.g., by Bernard et al., 2006). Respondents rated their agreement with each item on a 7-point Likert-type scale from 1 (*strongly disagree*) to 7 (*strongly agree*), and we calculated two estrangement scores by averaging responses to the items belonging to their respective subscales for atypical (5 items) and misfit (5 items).

### Results

#### Preliminary Analysis

Descriptive statistics and correlations are presented in Table 2. Examination of the means for the values of self-transcendence and self-enhancement for self and for perceptions of others' values suggests that people see themselves as more self-transcendence oriented (self-other Cohen's *d* = 0.72; Cohen, 1992) and less self-enhancement oriented (self-other Cohen's *d* = −0.68) than the typical British citizen.

**Table 2:** Descriptive statistics, Cronbach's alphas, and correlations for study 2 variables.

	**Mean**	**SD**	**Alpha**	**Voting**	**Natl. Vote**	**Loc. Vote**	**Oth Civ Bx**	**CEA**	**Atyp**	**Misfit**	**SE-Comp**.	**ST-Comp**.	**SE-Inst**.	**ST-Inst**.	**SE-Self**
Voting	0.82	0.36	0.79												
Natl Vote	0.83	0.39	–	0.87^***^											
Loc Vote	0.82	0.40	–	0.87^***^	0.61^***^										
Oth Civ Bx	0.36	0.27	0.79	0.28^***^	0.28^***^	0.23^***^									
CEA	3.52	0.64	0.85	0.22^***^	0.19^***^	0.20^***^	0.50^***^								
Atypical	3.50	0.63	0.80	−0.16^***^	−0.13^***^	−0.12^***^	−0.07^*^	0.24^***^							
Misfit	2.64	0.79	0.82	−0.12^***^	−0.10^**^	−0.10^**^	0.01	−0.09^**^	0.47^***^						
SE-Comp.	−0.15	0.68	0.78	−0.13^***^	−0.11^***^	−0.12^***^	−0.05	−0.10^**^	0.31^***^	0.24^***^					
ST-Comp.	0.03	0.56	0.85	0.11^***^	0.10^**^	0.09^**^	0.06	0.12^***^	−0.28^***^	−0.23^***^	−0.69^***^				
SE-Inst.	−0.16	0.78	0.84	−0.05	−0.05	−0.06	0.02	−0.02	0.19^***^	0.17^***^	0.29^***^	−0.18^***^			
ST-Inst.	0.15	0.64	0.91	0.03	0.03	0.04	−0.01	0.04	−0.17^***^	−0.18^***^	−0.21^***^	0.25^***^	−0.74^***^		
SE-Self	−0.73	0.78	0.80	−0.14^***^	−0.13^***^	−0.12^***^	−0.08^*^	−0.10^**^	−0.05	0.10^**^	0.32^***^	−0.18^***^	0.36^***^	−0.25^***^	
ST-Self	0.57	0.63	0.81	0.09^**^	0.09^**^	0.06^*^	0.22^***^	0.25^***^	−0.10^**^	−0.02	−0.13^***^	0.20^***^	−0.15^***^	0.23^***^	−0.62^***^

*** *p < 0.001*,

** *p < 0.01*,

* *p < 0.05. Voting mean of two voting items; Natl. Vote, voting in national election; Loc. Vote, voting in local election; Oth Civ Bx, other civic engagement behaviors; CEA, Civic Engagement Attitudes; Atyp, atypical subscale of cultural estrangement; SE-Comp, self-enhancement; ST, self-transcendence; Comp, Compatriot (perceived values of others); Inst, Institutions (perceived value encouragements)*.

Before turning to tests of our hypotheses, we note that, as shown in Table 2, personally-held self-transcendence values were positively and significantly related to both voting and other (non-voting-related) forms of civic engagement. In contrast, personally-held self-enhancement values were significantly negatively correlated with both forms of engagement, corroborating previous research on values and civic engagement (Schwartz, 2010; Vecchione et al., 2015). Personally held values correlated with cultural estrangement in the same directions that perceived values of others and of institutions were expected to correlate with estrangement.

#### Hypothesis Testing

##### Perceived values of others and civic engagement (H1a and H1b)

Turning to our focal concern with the relationship between perceived values of others and civic engagement, as shown in Table 2, perceived others' self-transcendence values were positively linked to voting, replicating Study 1. In contrast to Study 1, which found no link between perceptions of others' self-enhancement and voting, here we observed a negative effect, in line with hypothesis 1b. We found a similar effect for civic engagement attitudes, which were linked to higher perceptions of others' self-transcendence and lower perceptions of others' self-enhancement values. However, neither measure of values related to engaging in other forms of civic behavior.

##### Perceived values of others, cultural estrangement, and civic engagement (H2a-H2d)

We also hypothesized that perceptions of others' self-transcendence would be negatively related to feelings of cultural estrangement. This was the case, as the more that people reported believing that their compatriots valued self-transcendence, the less they reported feeling either atypical or like a misfit. Conversely, the more that people believed others valued self-enhancement, the more they reported feeling atypical or a misfit. These results both support the expectations presented in hypothesis 2a and help establish that the path from the predictor to the proposed mediator is tenable.

Hypothesis 2b, which suggested that feelings of estrangement would be negatively correlated with civic engagement, was supported for all combinations of the atypical/misfit subscales and for civic engagement, except between misfit and other (non-voting) civic behaviors. In general, the more estranged people felt, the less civic engagement they reported. These findings also help establish the link between the proposed mediator and outcome.

We therefore next tested the mediation hypothesis (H2c) that feeling estranged helps to explain the link between perceived values of others and civic engagement. For this analysis, we recognized that perceptions of both self-transcendence and self-enhancement had associations with both the mediator(s) and the outcome that were of approximately equal size but opposite in direction, thereby satisfying a key assumption behind the proper employment of a difference score (Edwards, 2002). Thus, we created a single score representing the relative strength of perceived others' self-transcendence values over self-enhancement (ST-SE; for similar uses of relative value strength indicators, see, e.g., Prentice and Sheldon, 2015). We tested a model in which this ST-SE difference score was the predictor, voting was the outcome, and atypical and misfit estrangement were simultaneous mediators (Model 4; Hayes, 2013). Once the mediators were entered into the model with perceived others' values, the effect of the ST-SE difference score was reduced from *b* = 0.041, *SE* = 0.001, *t*_(994)_ = 4.10, *p* < 0.001, to *b* = 0.025, *SE* = 0.010, *t*_(994)_ = 2.42, *p* = 0.016. Of the aspects of estrangement, only atypical was a significant predictor of voting, *b* = −0.063, *SE* = 0.021, *t*_(994)_ = −3.02, *p* = 0.003, and bootstrapping the indirect effect indicated mediation by atypical, *b* = 0.011 [0.004, 0.020], *SE* = 0.004.^6^

##### Institutional values, cultural estrangement, and civic engagement (H3a and H3b)

Finally, we turned to our hypotheses that the values that people believe are encouraged by cultural institutions are linked to civic engagement and cultural estrangement. As seen in Table 2, the zero-order correlations between both civic engagement variables and perceived institutional self-transcendence or self-enhancement (without regard for institution type) were small and non-significant. However, these results may belie associations that differ in direction across the five institutions we used as targets. Thus, in separate analyses, we predicted civic engagement and cultural estrangement from the ST-SE difference score, institution type, and the interaction between these two variables. The tests of the main effects and interactions are presented in Table 3.

**Table 3:** Test statistics from predicting cultural estrangement and civic engagement behaviors and attitudes from perceived values encouraged by institutions, institution types, and their interaction, Study 2.

	**F**	***p***
**OTHER CIVIC ENGAGEMENT BEHAVIOR**		
Value	0.02	0.898
Institution	0.40	0.812
Value × Institution Type	2.74	0.027
**CIVIC ENGAGEMENT ATTITUDES**		
Value	0.85	0.356
Institution	0.46	0.767
Value × Institution Type	1.88	0.111
**VOTING**		
Value	3.22	0.073
Institution	0.49	0.746
Value × Institution Type	1.55	0.186
**CULTURAL ESTRANGEMENT**		
Value	54.61	<0.001
Institution	0.63	0.642
Value × Institution Type	3.26	0.012

**Figure 1:**
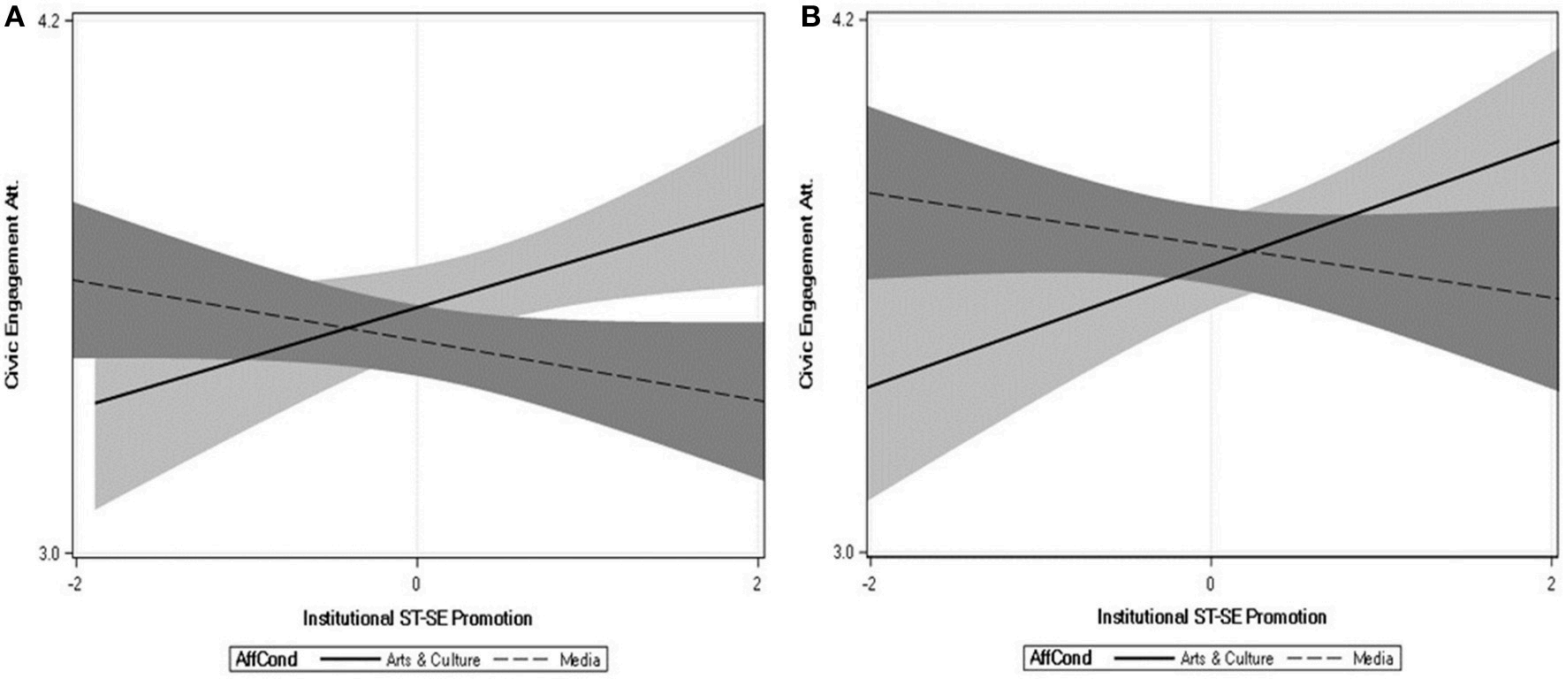
Relations between civic engagement attitudes (y axis) and the extent to which the institutions of Arts and Culture and Media are perceived to promote relatively more self-transcendence vs. self-enhancement values (x axis) in the UK **(A)** and the US **(B)**. Shaded regions reflect the 95% confidence regions around the regression lines.

**Figure 2:**
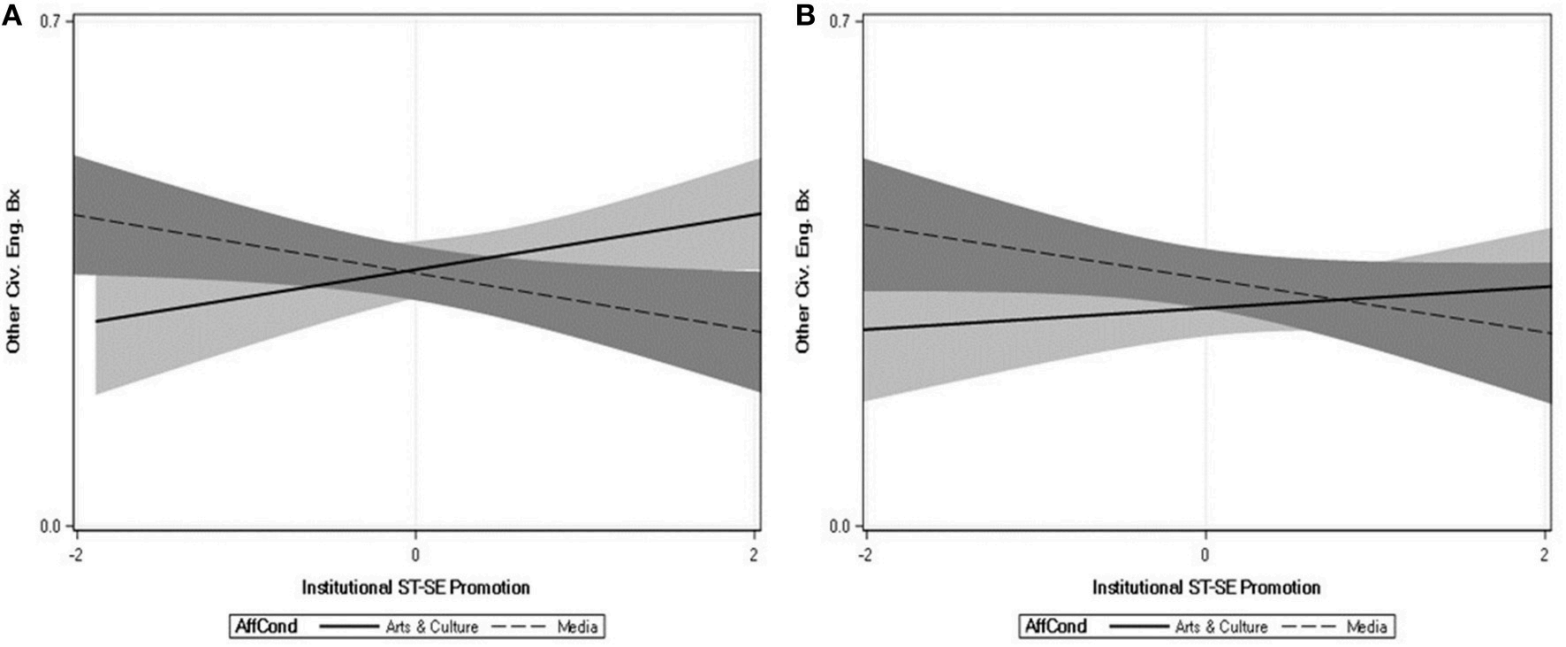
Relations between other (non-voting) civic engagement behaviors (y axis) and the extent to which the institutions of Arts and Culture and Media are perceived to promote relatively more self-transcendence vs. self-enhancement values (x axis) in the UK **(A)** and the US **(B)**. Shaded regions reflect the 95% confidence regions around the regression lines.

Here we highlight three key findings. First, perceiving institutions to encourage self-transcendence over self-enhancement was associated with lower estrangement. Second, this effect on cultural estrangement was qualified by a significant interaction between perceived institutional ST-SE values and institution type. All effects were negative. The strongest effect was within the government condition, *b* = −0.269, *SE* = 0.046, *t*_(889)_ = −5.78, *p* < 0.001, while the weakest effect was in the media condition, *b* = −0.057, *SE* = 0.041, *t*_(889)_ = −1.40, *p* < 0.162 (see Figure 1). Third, there was also a perceived institutional ST-SE × institution type interaction in the prediction of other civic engagement behaviors. This interaction appeared to be driven by the difference in slopes between the arts and culture and the media conditions, as displayed in Figure 2. To the extent that arts and culture institutions were perceived to encourage ST over SE values, civic engagement increased, *b* = 0.038, *SE* = 0.027, *t*_(889)_ = 1.75, *p* = 0.080, but as media institutions were perceived to encourage ST over SE values, civic engagement decreased, *b* = 0.040, *SE* = 0.018, *t*_(889)_ = −2.20, *p* = 0.028.

In summary, the media's and the government's promotion of relatively more self-enhancement over self-transcendence is linked to more estrangement and less engagement, whereas the promotion of self-transcendence over self-enhancement by arts and culture is linked to greater engagement.

### Brief Discussion

Study 2 replicates and extends Study 1 in several ways. First, it provides further support for H1, that perceptions of others' self-transcendence values, this time in relation to “a typical British person,” are positively related to voting and a range of civic engagement behaviors. Perceptions of others' self-enhancement values were also negatively related to voting (unlike in Study 1) but were unrelated to civic engagement behaviors other than voting (e.g., volunteering, donating money, signing petitions). We obtained these findings with an extended measure of Schwartz's (1992, 1994) values, giving us more reliable evidence in support of H1b than we had in Study 1, where we used single-item measures of self-transcendence and self-enhancement values.

Further, perceptions of others' values were related to cultural estrangement (H2a), and cultural estrangement was related to civic engagement (H2b), with results for self-transcendence and self-enhancement in the expected directions. Tests of our mediation hypothesis (H2d) revealed that not only did estrangement serve as a mediator of the relationship between perceptions of others' values and voting, but its explanatory power was specific to the atypicality aspect of cultural estrangement. Of the two subscales, misfit and atypicality, only atypicality seems to be a fairly direct reflection of feeling that one's own values are (un)like others.

Both Studies 1 and 2 were conducted with large, nationally representative British samples, but it remains to be seen whether similar patterns of results would occur in another culture. For this reason, we replicated the procedure and measures of Study 2 in a third study, this time using a sample based in the US.

## Study 3

### Material and Methods

#### Participants

The sample was obtained in April 2015 by Ipsos MORI, which collected a representative sample of the US population in terms of age, gender, region of the US, level of education, and income. The sample obtained had *N* = 1,005 and was 51.1% female. As in Study 2, age was binned by decade after a grouping to include 18–24 year olds, and every decade from 25 to 64 comprised at least 15% of the sample. Participants received points for the Ipsos benefit system in exchange for completing the survey.

#### Procedure

The procedure was identical to that of Study 2.

#### Measures

The measures were the same as those in Study 2, with some differences in wording to reflect the American sample. Scale descriptive statistics and zero-order correlations are provided in Table 4.

**Table 4:** Descriptive statistics, Cronbach alphas, and correlations for study 3 variables.

	**Mean**	**SD**	**Alpha**	**Voting**	**Natl. Vote**	**Loc. Vote**	**Oth Civ Bx**	**CEA**	**Atyp**	**Misfit**	**SE-Comp**.	**ST-Comp**.	**SE-Inst**.	**ST-Inst**.	**SE-Self**
Voting	0.75	0.41	0.87												
Natl vote	0.78	0.43	–	0.92^***^											
Loc vote	0.73	0.45	–	0.94^***^	0.74^***^										
Oth Civ Bx	0.33	0.28	0.83	0.34^***^	0.32^***^	0.33^***^									
CEA	3.73	0.68	0.88	0.25^***^	0.21^***^	0.25^***^	0.47^***^								
Atypical	3.45	0.70	0.82	−0.16^***^	−0.15^***^	−0.16^***^	−0.10^**^	−0.33^***^							
Misfit	2.89	0.88	0.86	−0.19^***^	−0.19^***^	−0.17^***^	0.02	0.01	−0.39^***^						
SE-Comp.	0.20	0.82	0.85	−0.04	−0.01	−0.06^*^	0.04	−0.05	0.35^***^	0.25^***^					
ST-Comp.	−0.17	0.65	0.87	0.07^*^	0.06	0.08^*^	−0.03	0.06	−0.37^***^	−0.29^***^	−0.78^***^				
SE-Inst.	−0.12	0.88	0.83	0.00	0.00	−0.01	0.09^**^	0.00	0.21^***^	0.15^***^	0.42^***^	−0.33^***^			
ST-Inst.	0.15	0.70	0.91	0.00	0.00	0.01	−0.04	0.04	−0.21^***^	−0.22^***^	−0.32^***^	0.37^***^	−0.72^***^		
SE-Self	−0.78	0.78	0.79	−0.07^*^	−0.04	−0.08^*^	−0.02	−0.11^***^	0.00	0.13^***^	0.12^***^	−0.04	0.28^***^	−0.22^***^	
ST-Self	0.59	0.60	0.83	0.10^**^	0.09^**^	0.10^**^	0.18^***^	0.22^***^	−0.12^***^	−0.05	0.10^**^	−0.06	−0.08^*^	0.18^***^	−0.59^***^

*** p < 0.001,

** p < 0.01,

* *p < 0.05. Voting, mean of two voting items; Natl. Vote, voting in national election; Loc. Vote, voting in local election; Oth Civ Bx, other civic engagement behaviors; CEA, Civic Engagement Attitudes; Atyp, atypical subscale of cultural estrangement; SE-Comp., Self-Enhancement; ST, self-transcendence; Comp, Compatriot (perceived values of others); Inst., Institutions (perceived value encouragements)*.

The results of an exploratory factor analysis on the civic engagement behavior items revealed a two-factor solution that was quite like that found in the UK sample. We used the same indicators as in Study 2 to create the same variables for voting (mean standardized loading = 0.87) and other civic engagement behaviors (mean standardized loading = 0.54).

The PVQ items for perceptions of compatriots and institutions again showed good fit to a clustered circumplex, so we again created the self-transcendence and self-enhancement scores for the perceived compatriot and institution formats of the questionnaires as in Study 2.

### Results

#### Preliminary Analysis

As in the British sample in Study 2, Americans saw themselves as more self-transcendence oriented (self-other Cohen's *d* = 0.72) and less self-enhancement oriented (self-other Cohen's *d* = −0.68) than the typical American citizen. Further, and as in Study 2, personally-held self-transcendence values were positively and significantly related to both voting and other (non-voting-related) forms of civic engagement. In contrast, personally-held self-enhancement values were significantly negatively correlated with voting but were unrelated to other forms of civic engagement (see Table 4).

#### Hypothesis Testing

##### Perceived values of others and civic engagement (H1a and H1b)

As reported in Table 4 people's perceptions of their compatriots' self-transcendence values were positively correlated with voting, but not with other forms of civic engagement; replicating the findings of Study 2. In contrast to Study 2 however, Americans' perceptions of their compatriots' self-enhancement were related only to voting in local elections, and neither type of perceived values was significantly tied to Americans' civic engagement attitudes.

Results in Table 4 also indicated that the more that people reported believing that their compatriots valued self-transcendence, the less they reported feeling atypical and like a misfit. Conversely, the more that people believed others valued self-enhancement, the more they reported feeling atypical and like a misfit. These findings replicate Study 2 and again establish that the path from the predictor to the proposed mediator is tenable.

##### Perceived values of others, cultural estrangement, and civic engagement (H2a-H2d)

In addition, the more estranged people felt, the less civic engagement they reported. However, as in Study 2, the misfit subscale did not relate to other civic behaviors.

We next tested the mediation hypothesis that feeling estranged helps to explain the link between perceived values of others and civic engagement. As in Study 2, both perceived compatriot self-transcendence and self-enhancement had associations with both the mediator(s) and outcome that were approximately equal in size and opposite in direction, allowing us to employ a difference score. We therefore entered this perceived compatriot ST-SE difference score as the predictor, voting as the outcome, and atypical and misfit as simultaneous mediators. Once the mediators were entered into the model, the association between perceived others' ST-SE and voting was reduced from *b* = 0.017, *SE* = 0.009, *t*_(991)_ = 1.82, *p* = 0.069, to *b* = −0.009, *SE* = 0.010, *t*_(991)_ = −0.90, *p* = 0.367. Of the aspects of estrangement, both atypical and misfit were unique, significant predictors of voting, *b* = −0.066, *SE* = 0.021, *t*_(991)_ = −3.20, *p* = 0.001 and *b* = −0.072, *SE* = 0.016, *t*_(991)_ = −4.63, *p* < 0.001, respectively. The bootstrapped indirect effects for atypical, *b* = 0.013 [0.005, 0.021] and for misfit, *b* = 0.013 [0.008, 0.020] did not contain zero, indicating that both forms of cultural estrangement mediated the link between voting and perceptions of others' values.

##### Institutional values, cultural estrangement, and civic engagement (H3a and H3b)

Finally, we conducted the same analyses concerning perceptions of institutional values that we had done in Study 2. The tests of the main effects and interactions across dependent variables are presented in Table 5. Here we highlight three key findings. First, there was again a strong, negative main effect of perceived institutional ST-SE on cultural estrangement, replicating Study 2. Second, and unlike Study 2, this effect was not qualified by a significant interaction between ST-SE and institution type. Third, there was a significant interaction between perceived institutional ST-SE and institution type in the prediction of civic engagement attitudes. This interaction appeared to be driven in part by the difference in slopes between the arts and culture and the media conditions, displayed in Figure 1 (right panel). As arts and culture institutions were perceived to encourage ST-SE, civic engagement attitudes were more positive, *b* = 0.136, *SE* = 0.051, *t*_(985)_ = 2.67, *p* = 0.008, but as media institutions were perceived to encourage ST-SE, civic engagement attitudes decreased, *b* = −0.059, *SE* = 0.049, *t*_(985)_ = −1.21, *p* = 0.223. Hence, as in Study 2, the perceived promotion of ST-SE by arts and culture is linked to more positive civic engagement attitudes, whereas the perceived promotion of these values by the media has the opposite effect. Finally, although the interaction was not significant, we present the relations between values and non-voting engagement behaviors for the media and arts in Figure 2 (right panel). As is clear in the figure, the patterns are fairly consistent with those observed in the British sample (Study 2), even though they did not reach significance in this American sample.

**Table 5:** Test statistics from predicting cultural estrangement and civic engagement behaviors and attitudes from perceived values encouraged by institutions, institution types, and their interaction, Study 3.

	***F***	***p***
**OTHER CIVIC ENGAGEMENT BEHAVIOR**		
Value	3.71	0.054
Institution	0.90	0.466
Value × Institution	0.83	0.505
**CIVIC ENGAGEMENT ATTITUDES**		
Value	3.49	0.062
Institution	2.28	0.059
Value × Institution	4.25	0.002
**VOTING**		
Value	0.25	0.620
Institution	1.06	0.376
Value × institution type	0.48	0.747
**CULTURAL ESTRANGEMENT**		
Value	63.53	<0.001
Institution	1.40	0.232
Value × institution type	1.15	0.330

### Brief Discussion

The primary aim of Study 3 was to examine to what extent the patterns we uncovered in a British context between perceived values of others and civic engagement also held in an American context. Although some of the relations between perceived values and civic engagement appeared weaker in the US than in the UK, there was a considerable amount of overlap between results in the two samples. For example, the more both Americans and Brits reported believing their average compatriots valued self-transcendence, the more they themselves reported voting and the less they reported feeling culturally estranged.

## General Discussion

The studies reported here, which were conducted in large, nationally representative samples across the UK and US, offer two main contributions to the existing literature: first, they show that perceptions of others' values have a consistent relationship with civic engagement, and, second, they indicate that cultural estrangement is a key mechanism that explains this relationship.

These novel findings may have important implications for the many groups working to increase civic engagement. In some ways, the presentation of the results so far, with many apparently small effect sizes, may belie their practical meaning. Here we illustrate how perceptions are related to odds of voting. As noted in section Preliminary analysis, the average British citizen reports being less self-enhancement oriented than they believe their average compatriot to be, Cohen's *d* = 0.68, and in raw scale values the gap is 0.58 (i.e., from mean self-enhancement actual = −0.15 and mean self-enhancement perceived = −0.73 in Table 2). Now let us examine the simple case of the prediction of voting in the national election from perceptions of compatriot values for self-enhancement. A logistic model of voting by perceived compatriot self-enhancement reveals an effect of perceived self-enhancement of −0.46, which means that for every 1 point increase on the scale of perceived compatriot self-enhancement, odds of voting decrease by 37% [37 = 1—exp(−0.46)]. What if the average citizen closed the gap by bringing their perceptions in line with what others actually self-report for self-enhancement? That is, what if the average citizen decreased the perception of self-enhancement by 0.58? The change in the log odds for closing this gap is given by completing the logistic equation with intercept and slope above for values of actual (−0.15) and perceived (−0.73) self-enhancement. The log odds difference is 0.27, which gives an odds increase for voting of 31%. This, in turn, translates to a 3.4% increase in overall turnout, or 2.23 million more voters (given the mid-2016 population estimate of 65.65 million; Office of National Statistics, 2017).

### Perception of Others' Values and Civic Engagement

Our primary finding, consistent across studies, was that individuals who thought that self-transcendence was valued by British culture (Study 1), by “a typical British person” (Study 2), and by “a typical American person” (Study 3) were more likely to report having voted in recent elections.

Across these studies, we found evidence that voting is more positively related to perceived others' self-transcendence (H1a) than that it is negatively related to perceived others' self-enhancement values (H1b). One possible explanation for why we see more robust (beneficial) effects of self-transcendence than (harmful) effects of self-enhancement is that care for others' welfare (reflected in self-transcendence values) is a motive more obviously associated with political and civic engagement than self-interest (as reflected in self-enhancement values) is associated with civic *dis*engagement. That is, people might have a number of different motives for their own political disengagement, including disillusionment (e.g., “All the parties sound the same”), low political outcome efficacy (e.g., “My vote doesn't count”), and low trust in the electoral and democratic system (e.g., “The system is rigged”). It could also be the case that people do not necessarily associate others' self-interested motives with disengagement. They might rather assume that their self-interested compatriots *would* get civically involved, but just in the pursuit of selfish rather than group interest. These questions would be fascinating to explore in future research, as there is little understanding at present of how individuals' expectations of others' values predict their civic engagement.

A second outcome of interest concerned civic engagement attitudes, more broadly (that is, not only relevant to voting). Results for this outcome were rather inconsistent across studies. Namely, when we measured civic engagement attitudes in Study 2, we found that the more British participants perceived other British people to care about self-transcendence, or to not care about self-enhancement, the more positive their attitudes were toward civic engagement. However, these findings did not hold with American citizens in Study 3. This might be because the US population is much larger and, arguably, more politically polarized than the UK. Research suggests that Americans consistently overestimate polarization in the attitudes of Democrats vs. Republicans, and they are more likely to fall prey to that overestimation if they themselves are politically engaged (Westfall et al., 2015). When Democrats and Republicans see themselves as so clearly opposed, the concept of a “typical compatriot's values” at a national level may be too broad, or perhaps too conflicted, to be associated with attitudes toward civic engagement.

Our hypotheses regarding perceptions of others' values were not supported when predicting civic engagement behaviors other than voting. One possible reason for these different patterns for voting vs. other forms of civic engagement may be the effort involved in each type of activity. It does not take much to claim community is important or to vote during broadly orchestrated elections. In contrast, many of the behaviors in the measure of other civic behaviors (e.g., volunteering, demonstrating, donating money) may require some cost in terms of time, money, or social reputation.

There might also be some types of civic engagement that are more likely to be affected by perceptions of others. For instance, perceptions could play a stronger role in behaviors that are collective and involve direct interaction with other people, such as joining a community campaign group, compared to behaviors that are explicitly solo activities, for example signing an online petition. It would be a good direction for future research to explore differences between such sub-classifications of civic engagement (cf. Corning and Myers, 2002).

Why might perceptions of others' values be associated with civic engagement? Considering the link between self-enhancement and disengagement, we revisit the notion that where there is a perceived norm of self-interest, people are motivated to fit in with that (Miller, 1999; Miller and Prentice, 2016). This same norm-following logic can also be marshaled to explain the reverse (and, as we found, stronger) link between perceptions of others' self-transcendence values and engagement: there is also a strong norm of benevolence in the cultures we sampled and that may be shared universally (Saucier, 2017), and people are motivated to act in line with that norm as well.

Of course, it may also be the case that actions influence values. For example, Bem (1972) proposed that individuals observe their own behavior and then infer what has caused them to act. If people observe that they have not voted, for instance, they might explain this with reference to others' values, e.g., “I didn't vote because I don't think I'm living in a society where people care about the welfare of others.” Similarly, Heider's (1958) consistency theory might suggest that people change their goals and values to be consistent with their volitional behavior *post facto*. In both of these explanations we assume that behavior is affected by cues in the environment. Of course, we cannot exclude other possibilities of causation. It could be that individuals project their own values onto others and assume, for example, that if they themselves care about co-operation then other people must too; this projection may then influence civic engagement, feelings of cultural estrangement, etc. It could also be that fit is important, such that outcomes like civic engagement are a function of the alignment between personal and perceived values (cf. Bleidorn et al., 2016). We have placed less emphasis on the projection and fit explanations simply because they are more difficult to address empirically, not because they are necessarily less likely. As such, probing the direction(s) of causality for the associations we observed provides one important and interesting avenue for future research.

### Cultural Estrangement Explains the Link Between Perceived Values of Others and Engagement

Few studies have addressed whether the perception of certain values, in particular, are associated with feelings of estrangement, often conceptualized as the comparison between one's own values and those of society and/or a rejection of dominant values (Bernard et al., 2006). We reasoned that the perception that others prioritize the welfare of the group (perceiving others' self-transcendence values), would be associated with lower estrangement and the perception that others prioritize self-interested goals would be associated with higher estrangement. This hypothesis (H2a) was consistently supported across the studies. We also found consistent evidence in support of H2b, that people were less likely to vote if they felt alienated (Study 1), atypical, or like a misfit (Studies 2 and 3). Feeling atypical (but not like a misfit) was also found to relate negatively to civic engagements other than voting. Further, our mediational hypothesis (H2d) was supported, in that feeling atypical (but not a misfit) helped explain the relationship between perceptions of compatriots' values and voting. These findings regarding atypicality in particular suggest that when individuals feel themselves to be a stranger, in terms of values, they are more likely to be disengaged from civic activities.

Taken together, these findings suggest that cultural estrangement may be an important mechanism by which perceptions of others' values are associated with levels of civic engagement. If people not only perceive others to be relatively high in self-transcendence vs. self-enhancement, but also feel that they are themselves fairly typical of their national values (as captured by lower scores on cultural estrangement), then perhaps they will be more likely to vote.

### Perceptions of Institutions' Values Are Also Linked to Civic Engagement

Along with exploring the consequences of perceiving other individuals' values, we also explored value perceptions regarding five types of institutions: arts and culture, education, government, media, and business.

As far as we know, no study has looked at the perceptions of the values that are encouraged by large, national, cultural institutions, nor how those perceptions might relate to civic engagement, yet these kinds of perceptions are fascinating reflections of how people view the societies in which they live. Although there was no general relationship between civic engagement and perceptions of the values that institutions in general promote, some significant results were evidenced for certain institutions. Specifically, civic engagement was positively associated with perceived promotion of self-transcendence by arts and culture institutions but negatively correlated with perceived promotion of self-transcendence by the media. These patterns might suggest that people are sensitive to a type of institutional value fit, such that when institutions are perceived as promoting certain expected values, civic engagement is higher. Though speculative and abstract, arts and culture institutions may be more likely to *talk with* the public in public spaces, rely on participation by community members, and “cross-promote” other community goings-on. Thus, this might explain why a perceived self-transcendent message from the arts is associated with greater civic involvement. In contrast, the media may be more likely to *talk at* the public in intrusive ways, inspire interindividual competition, and crowd out community with self-enhancement-promoting commercials. Thus, a self-enhancement message from the media would similarly seem to follow from the other tendencies associated with the institution, and a self-transcendent message would seem hypocritical.

### Limitations and Avenues for Future Research

One limitation of the present research arises from the retrospective self-report methodology we used to assess civic behaviors. In the absence of observed civic behavior or participant-matched voting data, we cannot know for certain that our participants were as civically engaged as they said. In general, however, it does not appear that socially desirable responding provides an alternative explanation for the results we observed (see footnote 2).

A significant limitation of the present set of studies, as alluded to in section Perception of Others' Values and Civic Engagement, is that the causality question remains unresolved: Do perceptions of others' values lead to civic engagement, or is some other causal pathway involved? We argue that these two variables are likely to have mutual influence on one another (possibly in a feedback loop), but this proposal requires an empirical investigation beyond the scope of this paper.

In the future, researchers could explore potential causal relationships between perceptions of others' values and civic engagement through experimental research where one or the other variable is manipulated (see section Cultural Estrangement Explains the Link Between Perceived Values of Others and Engagement). Ideally such studies would be conducted with samples of the general public or with specific types of organizations working to increase civic engagement. A research team in New Zealand (Harré et al., 2017) has suggested that people seeking social change tend to carry a so-called “tale of terror” about the values of their compatriots (i.e., that they care most about materialism, success, and status), but that this can be transformed to a “tale of hope” if people reflect instead that their compatriots value the common good. Harré et al. (2017) similarly suggest that campaigners will be more hopeful and therefore more effective if they can change their assumptions about what other people value.

Future research could also address the role of political ideology in the relationship between perceptions of others' values and different civic engagement outcomes. In the present paper we have measured a range of general civic outcomes, such as voting and demonstrating, that can encompass either conservative or liberal behaviors. Further, statistical control of demographic variables in the present data suffers from the issues outlined in the Appendix. Although it is a strength of this paper that we show connections between perceived values and civic outcomes, *regardless* of political ideology (as in Schwartz, 2010; Vecchione et al., 2015), this is a research avenue that deserves more attention, especially in disentangling how ideology informs values and vice versa.

It also remains to be seen whether these results would generalize beyond the two countries studied here. The US and UK have many cultural similarities and shared political histories, factors that could drive similar patterns among perceived values and civic engagement that may not hold outside of that particular cultural and historical/political overlap. In fact, within Europe, personally-held self-transcendence values are a significant predictor of political participation in countries with relatively old democratic traditions (e.g., Sweden) but not in post-socialist countries whose democratization is relatively recent (Slovenia and the Czech Republic; Fink, 2012). The relations between perceptions of others' values and civic engagement, then, might also vary by age and type of a nation's democracy, and should be explored in future research.

Another avenue for cross-cultural research is the question of if and why people systematically misperceive others as caring less about self-transcendence and more about self-enhancement than they do themselves. The literature on values discrepancy to date would seem to support the notion that this basic misperception is widespread, as it has previously been reported in Estonia (Dobewall and Strack, 2011), the UK (Bernard et al., 2006) and Russia (Lönnqvist et al., 2012).

In addition, it would be interesting to collect large enough samples to determine the relative incremental ability of one's own personal values and one's perceptions of others' values (and of cultural institutions' values) to predict outcomes. As noted in the Appendix, our current samples and measures were insufficient for this sort of analysis, but future projects could address this important question. In general, research should continue to examine the basic psychometric properties of the construct of perceived values of others and institutions. In the present work, multidimensional scaling indicated that perceived values may be structured quite similarly to personal values, and factor analysis provided preliminary evidence for distinct constructs. The convergent and discriminant validity of these constructs remains an especially important question for future research. Similarly, future research should seek to replicate the finding that estrangement may explain the perceived values-engagement link in light of the high potential for error-prone conclusions in the current study.

### Implications

Civic engagement is both a symptom and a driver of democratic processes and societal change. Whatever a citizen's definition of “better” may be, becoming civically engaged is fundamental to moving toward that ideal of a better society. Civic engagement is also fundamental to how many policy makers, politicians, and campaigners see change, i.e., that big and pressing issues such as climate change and economic justice will only be addressed if there is mass public participation, because top-down decision-making or technological solutions are insufficient (Common Cause Foundation, 2016).

Most of the research to date on civic engagement and values has focused on how personal value priorities relate to civic engagement (Schwartz, 2010; Pacheco and Owen, 2015; Vecchione et al., 2015). The present research indicates that perceptions of others' values also relate to voting and civic attitudes. These findings suggest that campaigners and policymakers might find it useful to encourage belief in the (accurate) norm that most people actually do care about the welfare of others more than self-interest; such a strategy could be implemented instead of or in addition to reminding people of their own self-transcendence values (Common Cause Foundation, 2016).

The present studies point to new avenues for research into the source of the “value-action gap” (e.g., Maio, 2010; see also Burford et al., 2015), which is the fact that abstract value priorities are not always associated with the behavioral outcomes one would expect to flow out of those values. It may be that, if people feel that their social environment affords them, via resources and support, opportunities to express self-transcendence values, then they may feel inclined to engage in actions consistent with their own self-transcendence values, e.g., to become civically involved. If, however, they feel that expression of their own self-transcendence values will be rebuffed by their environment, they may opt to not engage in behaviors consistent with their values. This raises the intriguing possibility that people who prioritize self-enhancement values might be more likely to act on their (presumably, relatively weak) self-transcendence values if they think they are living in an environment where these values can be expressed relatively easily.

Though still speculative, the applications of these results for policymakers and electoral campaigners are potentially deeper than simply encouraging people to believe in more accurate value norms. Instead, the results suggest that supporting and encouraging people's existing self-transcendence values could help to narrow the self-transcendence value-action gap. While such encouragement would presumably reduce feelings of cultural estrangement, another route would be to work on reducing estrangement directly and, in particular, the feeling of being atypical. On a national level this is clearly no mean feat, but one avenue for campaigners would be to be more explicit, through rhetoric and policies, of the ways in which the diversity of citizens is acknowledged and included. The concept of nationalism in both Britain and America has become increasingly associated with tribalism based on ethnic or class groups, and often for these reasons rejected by most but far-right political parties and citizens alike. It seems a healthy democracy might depend on building a widespread feeling among citizens that they belong in the civic group (i.e., the nation) enough to take part in civic matters.

We hope that other researchers continue the investigation of others' perceived values and cultural estrangement and the roles they may play in potentially important societal, interpersonal, and intrapersonal processes.

## Ethics Statement

Study 1

The Cardiff University School of Psychology ethics committee approved analysis of data (EC.15.04.14.4133R), and external organization YouGov followed recommendations for data collection with human subjects provided by the Framework for Research Ethics (FRE) as outlined by the Economic and Social Research Council (ESRC), and in compliance with the Data Protection Act, 1998. Participants gave their informed consent to take part in the study, in accordance with the Declaration of Helsinki.

Studies 2 and 3

Ipsos MORI collected data in full compliance with the ICC/ESOMAR Code of Conduct, the Data Protection Act 1998 and national research industry codes of conduct in the UK and USA (the Market Research Society and CASRO, respectively). Participants first completed informed consent and were assured of the confidentiality of their responses and their right to withdraw, in accordance with the Declaration of Helsinki.

## Author Contributions

RS led the preparation of this manuscript and designed, managed and supported the analysis of data in Studies 2 and 3. MP co-wrote this manuscript and led the analysis and reporting of data for all studies. LW and NW supported the design and analysis of Study 1 and provided feedback on the manuscript. TK and TC advised on the design, analysis, and interpretation of Studies 2 and 3, and provided feedback on the manuscript.

### Conflict of Interest Statement

RS, MP, LW, NW and TK declare that this work has been carried out without personal, professional, or financial relationships that could potentially be construed as a conflict of interest. TC works for the Common Cause Foundation, which secured the funding for collecting Studies 2 and 3.

## Footnotes

^1^American Psychological Association (2016). Available online at http://www.apa.org/education/undergrad/civic-engagement.aspx

^2^The voting question was written this way because social desirability might point one toward responding that “Yes I did my civic duty and voted” even if that were untrue. The phrasing employed was designed to lessen that social press in the hopes of obtaining more honest responses of non-voters.

^3^Specifically, participants completed the BIDR 6-Short (Bobbio and Manganelli, 2011), which has subscales for impression management (IM) and self-deceptive enhancement (SDE). Only SDE was related to our key behavioral civic engagement outcomes, and specifically only to voting (*r*_*UK*_ = 0.17, *r*_*US*_ = 0.09) Across Studies 2 and 3, we saw only one instance in which social desirability reduced to statistical non-significance an effect between values and engagement. Specifically, in Study 3, when we partialled SDE from the correlations between perceived values of others and voting, there was a negligible change in the correlation between perceived ST of others and voting in the US (from *r* = .07, *p* = 0.024, to *r*_*partial*_ = 0.06, *p* = 0.063). Because socially desirable responding had little impact on relations between values and civic outcomes, we do not refer to this variable further.

^4^Available online at www.europeansocialsurvey.org

^5^The items were: “I believe that I have a responsibility to help the poor and the hungry,” and “I am committed to serve in my community.” To members of the (primarily British) team, the phrase “the poor and the hungry” and the verb “serve” in this context did not feel reflective of contemporary British parlance.

^6^These mediation analyses are subject to many of the same potential pitfalls we outline in the Appendix if self and other values were to be treated simultaneously in the same model, but differ, in part, in that they were hypothesized. We therefore pursue the tests but discuss further under 5.4 Limitations and avenues for further research.

## Appendix

**Appendix: On Pursuing Tests of Incremental Validity for Self and Perceptions of Others' Values**

As noted in the introduction, readers might expect that we tested our main hypotheses after controlling for ratings of personally-held self-enhancement and self-transcendence values. We did not do so, however, for the following reasons.

In general, regression-based tests of unique prediction via simultaneous competitions for variance are fraught with inferential landmines. This is because “measurement unreliability and model misspecification will often have a deleterious and large effect on parameter estimates (and associated error rates) when covariates are entered into regression-based models. Consequently, under realistic assumptions, **it can be shown that a large proportion of incremental validity claims in many disciplines are likely to be false**” (Westfall and Yarkoni, 2016, p. 2; emphasis added). Said differently, in most realistic cases of regression-based analyses of incremental validity, the researcher runs a serious risk of conclusion errors, often via misattributing effects in regression-based models. If we were to conduct analyses testing whether, for example, perceptions of others' values added unique prediction of civic engagement after controlling for one's own personal values, we might mistakenly conclude evidence for the presence of incremental validity. Conversely, we might mistakenly conclude that perceived values of others do not have incremental validity over and above personally-held values. In the former case, we would be making a faulty assertion of incremental validity. In the latter, we might not pursue the results further because they would appear to lack novelty.

The key problem is that both problematic conclusions are often more likely than claims to either incremental or non-incremental validity that are sound. The key question is does the present research situation fit the profile for a case that is likely to lead to problematic conclusions? As readers will notice when reading the methods sections of Studies 2 and 3, we have large samples and conventionally acceptable measurement reliabilities, conditions that seem conducive to drawing sound conclusions from incremental tests via regression. However, it is under these conditions that researchers are often most likely to misattribute effects of simultaneous predictors in an attempt to make arguments for predictive utility (i.e., that there is an effect of a predictor even after controlling for a potential confound):

“even at a conventionally acceptable reliability of 0.7 or 0.8, the [Type I] error rate can still be extremely high if the sample size and/or indirect effect are large. The non-monotonic effect of reliability has a compound explanation that becomes clear when one considers each extreme separately. When reliability is very low … it becomes almost impossible to detect any effect. Conversely, when reliability is very high, the model is able to avoid misattributing the effect of the covariate to the predictor of interest. In the middle, however, there exists a territory where effects are large enough to afford detection, but reliability is too low to prevent misattribution.” (Westfall and Yarkoni, 2016, p. 5).

Similarly, in the present case, a related but different claim to be made on the basis of modeling is that self and other values are separately contributing constructs when they are both significant predictors in a simultaneous regression. Unfortunately, the statistical characteristics that make the argument for predictive utility so error prone, and that hold in the present case, largely hold for the separate contributions claim as well, and **the error rate for this claim approaches 100%** as sample sizes increase with modest reliabilities of 0.7–0.8 and small to moderate predictor intercorrelation (i.e., *r* = 0.3–0.5; cf. Figure 4A; Westfall and Yarkoni, 2016).

We can compute the probabilities for drawing conclusions about our parameters in an incremental test based on our observed correlations and reliabilities with the use of the incremental validity error rate calculator (“Ivy”) provided by Westfall and Yarkoni (2016; https://jakewestfall.shinyapps.io/ivy_app/). To take just one example, for self-enhancement values in Study 2, the probability of concluding both predictors have an effect is 74%, but this value includes the error rate that is inflated well over conventionally accepted levels of 5% and is likely over 50% (Figure 4A; Westfall and Yarkoni, 2016). In short, the large sizes of the current samples work counterintuitively in concert with the measured characteristics (i.e., the reliabilities and zero-order correlations) in ways that make confident conclusions that effects on voting are “just” because of perceptions, or “just” because of personal values, or are actually due to separate contributions of the two variables, highly error-prone.

The astute reader might further suspect that the situation could be improved by moving from regression to SEM, thereby accounting for measurement error. Unfortunately that is not the case presently. As the simulations in Westfall and Yarkoni (2016) demonstrate, we would need *N* = 1,600 (given our observed reliabilities) to have 80% power for detecting unique incremental effects (i.e., partial correlations) of 0.10, nearly as large as any incremental effect could be given the zero-order correlation patterns in Studies 2 and 3. The zero order correlations make it clear that we should not expect incremental effects of even that magnitude, given that those associations between predictors and voting are around or below 0.10 (cf. Figure 12B; Westfall and Yarkoni, 2016). In fact, *N* = 1,600 is a low value more reflective of the optimistic case for self-transcendence values, which are less-strongly correlated between self and perceived variables than self and perceived are for self-enhancement values. The situation for Study 3 is not substantially improved given the smaller zero order effects despite some slight gains in reliability overall.

In sum, are we justified in making the argument that perceived values are predictive of voting *beyond* the effect of personal values? No. However, we have not advanced that argument. Thus, we make no claims to incremental validity for perceived vs. self-values in the current paper, as we do not want to put ourselves in the position of making errant claims for or against incremental validity given the limits of the current studies for addressing such questions well. We do believe, however, that the project holds the possibility of providing incremental knowledge if it can demonstrate new types of relationships among the variables we explore.
